# Circulating erythroferrone has diagnostic utility for acute decompensated heart failure in patients presenting with acute or worsening dyspnea

**DOI:** 10.3389/fcvm.2023.1195082

**Published:** 2024-01-08

**Authors:** Sarah Appleby, Chris Frampton, Mark Holdaway, Janice Chew-Harris, Oi Wah Liew, Jenny Pek Ching Chong, Lynley Lewis, Richard Troughton, Shirley Beng Suat Ooi, Win Sen Kuan, Irwani Ibrahim, Siew Pang Chan, A. Mark Richards, Christopher J. Pemberton

**Affiliations:** ^1^ Department of Medicine, Christchurch Heart Institute, University of Otago Christchurch, Christchurch, New Zealand; ^2^ Department of Medicine, University of Otago Christchurch, Christchurch, New Zealand; ^3^ Cardiovascular Research Institute, National University of Singapore, Singapore, Singapore; ^4^ Department of Cardiology, Te Whatu Ora, Christchurch, New Zealand; ^5^ Emergency Department, National University Hospital, Singapore, Singapore

**Keywords:** erythroferrone, ERFE, acute decompensated heart failure, diagnosis, atrial fibrillation, obesity

## Abstract

**Objectives:**

In dyspneic patients with atrial fibrillation (AF) or obesity, the diagnostic performance of NT-proBNP for acute heart failure is reduced. We evaluated the erythroblast derived protein erythroferrone (ERFE) as an ancillary biomarker for the diagnosis of acute decompensated heart failure (ADHF) in these comorbid subgroups in both Western and Asian populations.

**Methods:**

The diagnostic performance of ERFE (Intrinsic Lifesciences) and NT-proBNP (Roche Cobas e411) for ADHF was assessed in 479 New Zealand (NZ) and 475 Singapore (SG) patients presenting with breathlessness.

**Results:**

Plasma ERFE was higher in ADHF, compared with breathlessness from other causes, in both countries (NZ; 4.9 vs. 1.4 ng/ml, *p* < 0.001) and (SG; 4.2 vs. 0.4 ng/ml, *p* = 0.021). The receiver operating characteristic (ROC) areas under the curve (AUCs) for discrimination of ADHF were reduced in the NZ cohort compared to SG for ERFE (0.75 and 0.84, *p* = 0.007) and NT-proBNP (0.86 and 0.92, *p* = 0.004). Optimal cut-off points for ERFE yielded comparable sensitivity and positive predictive values in both cohorts, but slightly better specificity, negative predictive values and accuracy in SG compared with NZ. In patients with AF, the AUC decreased for ERFE in each cohort (NZ: 0.71, *n* = 105, SG: 0.61, *n* = 44) but increased in patients with obesity (NZ: 0.79, *n* = 150, SG: 0.87, *n* = 164).

**Conclusions:**

Circulating ERFE is higher in patients with ADHF than in other causes of new onset breathlessness with fair diagnostic utility, performing better in Asian than in Western patients. The diagnostic performance of ERFE is impaired in patients with AF but not patients with obesity.

## 1 Introduction

Heart failure (HF) is characterized by the presence of multiple comorbidities which may contribute to its progression whilst potentially obscuring its diagnosis. Circulating levels of B-type natriuretic peptide (BNP) and its congener amino-terminal proBNP (NT-proBNP) are endorsed by international guidelines for the diagnosis and management of heart failure (1, 2). However, levels of these peptides are altered by comorbidities including atrial fibrillation (AF) and obesity (3–6) which can impair their diagnostic performance. For example, the AUC for NT-proBNP in the discrimination of HF among acutely breathless patients without AF is about 0.90 but is markedly decreased in the presence of AF to about 0.70 (4). Likewise, body mass index (BMI) has been shown to reduce the areas under the curve (AUCs) for both BNP and NT-proBNP in patients with obesity compared to non-obese patients (6). Thus, other markers that can complement or supplant NT-proBNP or BNP are required.

Erythroferrone (ERFE; previously described as FAM132B, myonectin, and CTRP15) is a hormone released from erythroblasts in stress induced erythropoiesis where it increases the availability of iron for new red blood cell synthesis to meet the demands of increased erythropoietic activity (7). The majority of ERFE literature concerns its role in iron homeostasis; however, there have been recent publications on the role of ERFE in cardiovascular disease (8–11). A cardioprotective role for ERFE has been demonstrated in mouse knock-out models subjected to an acute myocardial ischemic injury through the suppression of cardiomyocyte apoptotic and macrophage inflammatory responses (8). ERFE has also been reported to be down-regulated in the left ventricle during pressure-overload and as myocardial ERFE overexpression alleviated load-induced hypertrophic and fibrotic responses, ERFE may be a novel player countering overload-induced adverse cardiac remodelling (9). Thus, ERFE may partly mediate the cardiovascular benefits of endurance exercise and counter cardiac hypertrophy/fibrosis, making it a potential therapeutic target for the prevention or treatment of cardiac disease. ERFE has also been investigated in the diagnosis of coronary artery disease (CAD) with widely discrepant reports. Shokoohi Nahrkhalaji et al. showed serum ERFE was elevated in CAD patients compared to controls, with good discriminatory power for CAD [AUC of 0.855 (95% CI 0.808–0.902, *p* < 0.001)] (10). Conversely Zhang et al. found decreased levels of plasma ERFE in CAD patients with poor discrimination [AUC of 0.665 (95% CI 0.587–0.743, *p* < 0.001)] of CAD (11).

Circulating levels of ERFE in acute heart failure and its potential diagnostic application for ADHF have not been documented. Here we provide the first report evaluating plasma levels of ERFE in ADHF—in cohorts of both Western and Asian patients presenting with acute dyspnea to hospital emergency departments. We have compared its discriminatory powers to that of NT-proBNP.

## 2 Materials and methods

### 2.1 Study population

Patients were recruited at the Emergency Departments (EDs) in Christchurch Hospital, New Zealand (NZ; *n* = 479) and the National University Hospital, Singapore (SG; *n* = 475) according to matched recruitment criteria (12). Recruitment for the NZ cohort occurred from 2007 to 2019 and SG between 2010 and 2013. Shortness of breath as the primary complaint triggering presentation to the ED was the key inclusion criterion. Exclusion criteria included; under 18 years of age, shortness of breath related to trauma, patients with an acute ST-elevation myocardial infarction and those on haemodialysis for renal failure. This study was performed in compliance with the principles outlined in the Declaration of Helsinki. All patients gave written informed consent to participate in the studies. Study protocols were approved by the local ethics committee of the two institutions.

### 2.2 Adjudication

The adjudicated diagnosis of ADHF was made in accordance with the 2016 ESC taskforce guidelines (1), by two independent clinicians (an ED specialist and a cardiologist) with access to all clinical data whilst blinded to NT-proBNP and ERFE results. In NZ, disagreement between the two adjudicators was resolved by consensus and in SG, a third adjudicator gave a final diagnosis.

### 2.3 Clinical assessment and blood collection

Assessment included a comprehensive medical history, routine biochemical analyses, chest radiography, ECG recordings and pulse oximetry. Venous blood samples drawn at presentation were taken into EDTA collection tubes, the plasma rapidly separated, snap frozen at −80°C and biobanked at until assay.

### 2.4 Biomarker measurement

Human ERFE was measured using a commercially available ELISA kit (SKU# ERF-001, Intrinsic Lifesciences) and performed following the manufacturers’ instructions. All ERFE measurements were conducted at the Christchurch Heart Institute, New Zealand. The inter-assay coefficient of variation (CV) of low (5 ng/ml, 37%) and high (35 ng/ml, 36%) quality control samples, with intra-assay CVs at 28% and 25% were derived over 16 and 29 assays, respectively. NT-proBNP measurements were assessed using the commercially available Elecsys proBNP II assay, a chemiluminescent two-site assay conducted on the Roche Cobas e411 analyser (Roche Diagnostics GmbH, Mannheim, Germany). NT-proBNP had an inter-assay CV of 5.5% and 5.7% for the low (845 pg/ml) and high (4860 pg/ml) quality control samples, respectively. Haemoglobin measurements were determined at the core biochemistry laboratories of the respective institutions.

### 2.5 Statistical analysis

Values are expressed as median [interquartile ranges (IQRs)], or counts and percentages as appropriate. The Shapiro–Wilk test of normality was used to determine whether the data were normally distributed and where applicable, skewed data were normalised by log_10_-transformation. Pearson's correlation coefficient was calculated on log_10_ transformed variables to examine any relationships between ERFE concentrations and other continuous variables. *T*-tests were used for comparisons of continuous measures between the two cohorts. The prevalence of ADHF, AF and obesity were compared between the two cohorts using Chi-squared tests. Multiple linear regression analysis was used to identify any independent associations of demographic and clinical features to plasma ERFE concentrations within each cohort.

The overall discriminatory power of markers (ERFE and NT-proBNP) for the diagnosis of ADHF was analysed separately in NZ and SG patients and then further analysed after stratification for presence of AF and obesity. This was assessed using areas under the ROC curves (AUCs) derived from raw data for ERFE and NT-pro-BNP, and from binary logistic models using log_10_ transformed values after adjustment for significant demographic and clinical risk factors within each cohort. The sensitivity, specificity, positive (PPV) and negative predictive values (NPV) and accuracy of ERFE and NT-proBNP for the diagnosis of ADHF were calculated using ROC-derived optimal values at the point closest to perfect sensitivity and specificity (13). Pair-wise comparisons were used to determine if ROC curves were statistically different from each other. All statistical assessments were made using SPSS v26 (IBM Corp, Armonk, NY) and graphical representation using SigmaPlot v14.5 (Systat Software, San Jose, CA). In all analyses, a *p*-value of <0.05 was considered statistically significant.

## 3 Results

### 3.1 Clinical characteristics of patients

Data from a total of 954 patients were included in this analysis; (479 NZ and 475 SG). Characteristics for Western and Asian cohorts are presented in Table 1 and Supplementary Table S1. The NZ cohort consisted largely of NZ European patients (*n* = 430, 90%) with minorities of Māori (3%), Pacific Islander (0.8%) and other ethnicities (6%). The SG cohort was more ethnically diverse including Chinese (45%), Malay (29%), Indian (18%) and others (8%). As previously published (12), recruitment criteria were the same in both countries. As shown in Table 1, the two cohorts differed in age (younger in SG), renal function (reduced in NZ) and key elements of medical history. The adjudicated diagnosis of ADHF was higher in the NZ cohort (35.7%, *n* = 171) compared with SG cohort (24.2%, *n* = 115, *p* < 0.001). The overall prevalence of AF was more frequent in the NZ cohort (23.1%, *n* = 105) than the SG cohort (10.1%, *n* = 44, *p* < 0.001). Prevalence of obesity in Western patients (classified as BMI ≥ 30 kg/m^2^) (34%, *n* = 150) was lower than Asian patients (classified as BMI ≥ 27.5 kg/m^2^) (40%, *n* = 163, *p* = 0.039).

**Table 1:** Clinical characteristics of the breathless cohorts and ADHF subgroup.

	Whole cohort			ADHF		
	NZ (*n* = 479)	SG (*n* = 475)	*p*-value	NZ (*n* = 171)	SG (*n* = 115)	*p*-value
	Median (IQR)	Median (IQR)		Median (IQR)	Median (IQR)	
Age (years)	72 (62–81)	55 (45–63)	<0.001	78 (69–84)	58 (52–69)	<0.001
BMI (kg/m^2^)	26.9 (23.1–31.6)	26.1 (22.9–29.9)	0.091	26.9 (23.6–32.1)	27.0 (23.6–31.8)	0.975
eGFR (ml/min/1.73 m^2^)	61.0 (48.0–74.8)	88.8 (64.6–104.2)	<0.001	54.0 (41.0–65.0)	68.5 (45.1–86.9)	<0.001
SaO_2_ (%)	95 (93–97)	98 (96–100)	<0.001	96 (93–97)	97 (95–99)	<0.001
Heart rate (bpm)	92 (76–110)	89 (75–103)	0.040	90 (74–111)	94 (82–106)	0.701
Haemoglobin	136.0 (121.5–147.0)	136.0 (122.0–150.0)	0.478	128.0 (110.0–140.0)	128.0 (115.0–144.0)	0.062
ERFE (ng/ml)	2.4 (0.9–6.1)	0.71 (0.19–2.9)	<0.001	4.9 (2.4–11.0)	4.2 (1.7–8.0)	0.009
NT-proBNP (ng/L)	1035.1 (212.6–3903.8)	162.9 (38.8–2004.0)	0.007	4330.0 (1999.2–9015.2)	3637.0 (1804.0–8473.0)	0.511
CRP (mg/L)^a^	23 (7–79)	ND		13 (5–42)	ND	
WBC (×10^9^/L)	9.0 (7.1–12.1)	ND		7.9 (6.3–10.6)	ND	
	Count (%)	Count (%)	** **	Count (%)	Count (%)	** **
Gender (female)	194 (40.5%)	149 (31.4%)	0.004	59 (34.5%)	24 (20.9%)	0.013
ADHF	171 (35.7%)	115 (24.2%)	<0.001			
Medical history						
CHF	150 (31.3%)	70 (14.7%)	<0.001	85 (49.7%)	47 (40.9%)	0.129
MI	123 (25.7%)	73 (15.4%)	<0.001	62 (36.3%)	35 (30.4%)	0.308
CAD	210 (43.8%)	161 (33.9%)	0.001	97 (56.7%)	66 (57.4)	0.724
Hypertension	271 (56.6%)	248 (52.2%)	0.152	114 (66.7)	77 (67.0%)	0.986
CABG	53 (11.1%)	48 (10.1%)	0.674	31 (18.1%)	21 (18.3%)	0.977
PTCA	64 (13.4%)	49 (10.3%)	0.161	24 (14.0%)	21 (18.3%)	0.336
Diabetes	92 (19.2%)	158 (33.3%)	<0.001	43 (25.1%)	61 (53.0%)	<0.001
Hyperlipidaemia	216 (45.1%)	218 (45.9%)	1.000	83 (48.5%)	62 (53.9%)	0.487
Renal impairment	80 (16.7%)	36 (7.6%)	<0.001	43 (25.1%)	16 (13.9%)	0.022
COPD	191 (39.9%)	32 (6.7%)	<0.001	44 (25.7)	8 (7.0%)	<0.001
Liver disease	23 (4.8%)	13 (2.7%)	0.125	14 (8.2%)	3 (2.6%	0.050
Cancer	118 (24.6%)	20 (4.2%)	<0.001	46 (26.9%)	4 (3.5%)	<0.001
Arrhythmia	167 (34.9%)	51 (10.7%)	<0.001	84 (49.1%)	24 (20.9%)	<0.001
Symptoms and signs						
PND	208 (43.4%)	171 (36.0%)	0.008	100 (58.5%)	61 (53.0%)	0.320
Orthopnoea	289 (60.3%)	210 (44.2%)	<0.001	125 (73.1%)	80 (69.6%)	0.582
Dyspnea at rest	363 (75.8%)	280 (58.9%)	<0.001	137 (80.1%)	77 (67.0%)	0.012
Rales	261 (54.5%)	154 (32.4%)	<0.001	119 (69.6%)	91 (79.1%)	0.158
Rhonchi	62 (12.9%)	83 (17.5%)	0.058	11 (6.4%)	10 (8.7%)	0.472
Oedema	180 (37.6%)	104 (21.9%)	<0.001	109 (63.7%)	73 (63.5%)	0.964
ECG findings						
Normal	107 (22.3%)	173 (36.4%)	<0.001	18 (10.5%)	14 (12.2%)	0.730
Atrial fibrillation	105 (21.9%)	44 (9.3%)	<0.001	66 (38.6%)	26 (22.6%)	0.003
LBBB	26 (5.4%)	10 (2.1%)	0.010	14 (8.2%)	5 (4.3%)	0.180
Chest x-ray findings						
Normal	102 (21.3%)	224 (47.2%)	<0.001	8 (4.7%)	11 (9.6%)	0.113
Interstitial oedema	55 (11.5%)	55 (11.6%)	0.839	52 (30.4%)	38 (33.0%)	0.674
Pneumonia	56 (11.7%)	20 (4.2%)	<0.001	16 (9.4%)	6 (5.2%)	0.193

ND, no data available.

ADHF, acute decompensated heart failure; BMI, body mass index; eGFR, estimated glomerular filtration rate; SaO_2_, arterial oxygen saturation; ERFE, Erythroferrone; NT-proBNP, amino-terminal proBNP; CRP, C-reactive protein; WBC, white blood cell count; CHF, chronic heart failure; MI, myocardial infarction; CAD, coronary artery disease; CABG, coronary artery bypass grafting; PTCA, percutaneous transluminal coronary angioplasty; COPD, chronic obstructive pulmonary disease; PND, paroxysmal nocturnal dyspnea; LBBB, left bundle branch block.

^a^ Data available for 283/479 of the whole cohort and 91/171 for the ADHF subgroup.

### 3.2 ERFE concentrations

Overall median ERFE levels were significantly higher in dyspneic NZ vs. SG patients (2.4 [IQR 0.9–6.1] ng/ml vs. 0.71 [IQR 0.19–2.9] ng/ml, *p* < 0.001). ERFE and NT-proBNP levels were not normally distributed in either population and were log_10_ transformed prior to analyses. Plasma ERFE correlated with several clinical variables in both populations (Table 2), with the strongest associations being to NT-proBNP (NZ: *r* = 0.43, SG: *r* = 0.57, both *p* < 0.001), haemoglobin (NZ: *r* = −0.39, SG: *r* = −0.30, both *p* < 0.001) and to normal chest x-ray (CXRnorm) in SG (*r* = −0.44, *p* < 0.001).

**Table 2:** Correlations of log_10_ERFE with clinical variables.

	New Zealand		Singapore	
Clinical variable	Correlation coefficient (Pearsons r)	Significance (*p*-value)	Correlation coefficient (Pearsons r)	Significance (*p*-value)
Log_10_NT-proBNP	0.43	<0.001	0.57	<0.001
Haemoglobin	−0.39	<0.001	−0.30	<0.001
Haematocrit	−0.33	<0.001	ND	ND
CXRnorm	−0.21	<0.001	−0.44	<0.001
CXRinoed	0.18	<0.001	0.21	<0.001
Age	0.18	<0.001	0.22	<0.001
BMI	0.14	0.003	0.03	0.612
History of hypertension	0.14	0.002	0.17	<0.001
CAD	0.16	0.001	0.17	<0.001
MI	0.12	0.007	0.09	0.049
CHF	0.27	<0.001	0.27	<0.001
COPD	−0.11	0.020	0.15	0.001
Gender	0.02	0.687	0.04	0.416
Arrhythmia	0.21	<0.001	0.20	<0.001
Diabetes	0.03	0.551	0.20	<0.001
Hyperlipidemia	0.05	0.271	−0.02	0.736
PND	0.07	0.118	0.10	0.026
Orthopnoea	0.14	0.003	0.21	<0.001
Rales	0.26	<0.001	0.36	<0.001
Atrial fibrillation	0.22	<0.001	0.18	<0.001
eGFR	−0.22	<0.001	−0.29	<0.001
Ejection fraction	−0.10	0.256	ND	ND
Respiration rate	0.04	0.385	0.18	<0.001
Log_10_CRP^a^	0.21	<0.001	ND	ND
WBC	0.01	0.919	ND	ND

ND, no data available.

NT-proBNP, amino-terminal proBNP; CXRnorm, presence of a normal chest x-ray; CXRinoed, presence of interstitial oedema on chest x-ray; BMI, body mass index; CAD, coronary artery disease; MI, myocardial infarction; CHF, chronic heart failure; COPD, chronic obstructive pulmonary disease; PND, paroxysmal nocturnal dyspnea; eGFR, estimated glomerular filtration rate; CRP, C-reactive protein; WBC, white blood cell count.

^a^ Data available for 283/479.

Putative predictors were assessed for independent associations with plasma ERFE in multivariable analyses conducted separately in each cohort, incorporating age, gender, BMI, height, haemoglobin, eGFR, ejection fraction (NZ only), presence of AF on ECG, history of hypertension, myocardial infarction (MI), chronic heart failure (CHF), chronic obstructive pulmonary disease (COPD) and CAD (Table 3). In both countries, pulmonary rales and low haemoglobin levels were independent predictors of increased plasma ERFE and presence of a normal chest x-ray with lower levels of plasma ERFE. Independent associates of ERFE that differed between the two countries were BMI and AF in the NZ cohort, and presence of arrhythmia, orthopnoea and diabetes in the SG cohort. These models generated adjusted *r*^2^ values of 0.27 and 0.29 for NZ and SG, respectively, indicating most (70%–75%) of inter-individual variation in ERFE is determined by other factors not measured within this study. Table 4 lists the variables remaining independently associated with the diagnosis of ADHF after multivariable analysis for the two cohorts separately. Plasma ERFE, NT-proBNP and the presence of interstitial oedema on chest x-ray (CXRinoed) were the only variables in common to both cohorts. Four further variables were significant in the NZ cohort (COPD, normal chest x-ray, paroxysmal nocturnal dyspnea and AF) with three others in SG (history of CHF, orthopnoea and pulmonary rales).

**Table 3:** Independent associates of plasma ERFE.

	New Zealand^a^			Singapore^b^		
	Standardized coefficients			Standardized coefficients		
	Beta	t	*p*-value	Beta	t	*p*-value
CXRnorm	−0.139	−2.994	0.003	−0.298	−6.456	<0.001
Haemoglobin	−0.328	−7.469	<0.001	−0.182	−4.231	<0.001
Rales	0.170	3.631	<0.001	0.139	2.952	0.003
BMI	0.155	3.534	<0.001			
AF	0.135	3.053	0.002			
Orthopnoea				0.108	2.468	0.014
Arrhythmia				0.113	2.685	0.008
Diabetes				0.087	2.035	0.042

CXRnorm, presence of a normal chest x-ray; BMI, body mass index; AF, atrial fibrillation.

^a^ NZ *r*^2 ^= 0.27.

^b^ SG *r*^2 ^= 0.29.

**Table 4:** Independent associates of acute decompensated heart failure.

					95% C.I. for OR	
	B	S.E.	Sig.	OR	Lower	Upper
OR New Zealand cohort (*n* = 479)						
Log_10_ ERFE	0.924	0.302	0.002	2.518	1.393	4.554
Log_10_ NT-proBNP	1.572	0.337	<0.001	4.816	2.488	9.322
CXRinoed	3.459	0.810	<0.001	31.775	6.499	155.346
COPD	−1.273	0.351	<0.001	0.280	0.141	0.557
CXRnorm	−1.107	0.476	0.020	0.330	0.130	0.841
PND	1.334	0.343	<0.001	3.798	1.937	7.446
ECGafib	0.820	0.373	0.028	2.271	1.093	4.722
Constant	−7.100	1.246	<0.001	0.001		
Singapore cohort (*n* = 475)						
Log_10_ ERFE	0.645	0.262	0.014	1.906	1.141	3.184
Log_10_ NT-proBNP	1.181	0.285	<0.001	3.257	1.862	5.696
Cxinoed	1.183	0.453	0.009	3.266	1.344	7.934
CHF	0.824	0.407	0.043	2.279	1.027	5.058
Orthopnoea	0.911	0.369	0.014	2.486	1.206	5.124
Rales	1.566	0.368	<0.001	4.789	2.328	9.850
Constant	−6.517	0.975	<0.001	0.001		

CI, confidence interval; OR, odds ratio; COPD, chronic obstructive pulmonary disease; CXRnorm, presence of a normal chest x-ray; CXRinoed, presence of interstitial oedema on chest x-ray; PND, paroxysmal nocturnal dyspnea; ECGafib, presence of AF on ECG; CHF, chronic heart failure; ERFE, erythroferrone; NT-proBNP, amino-terminal proBNP.

### 3.3 ERFE in diagnosis of ADHF

**Figure 1:**
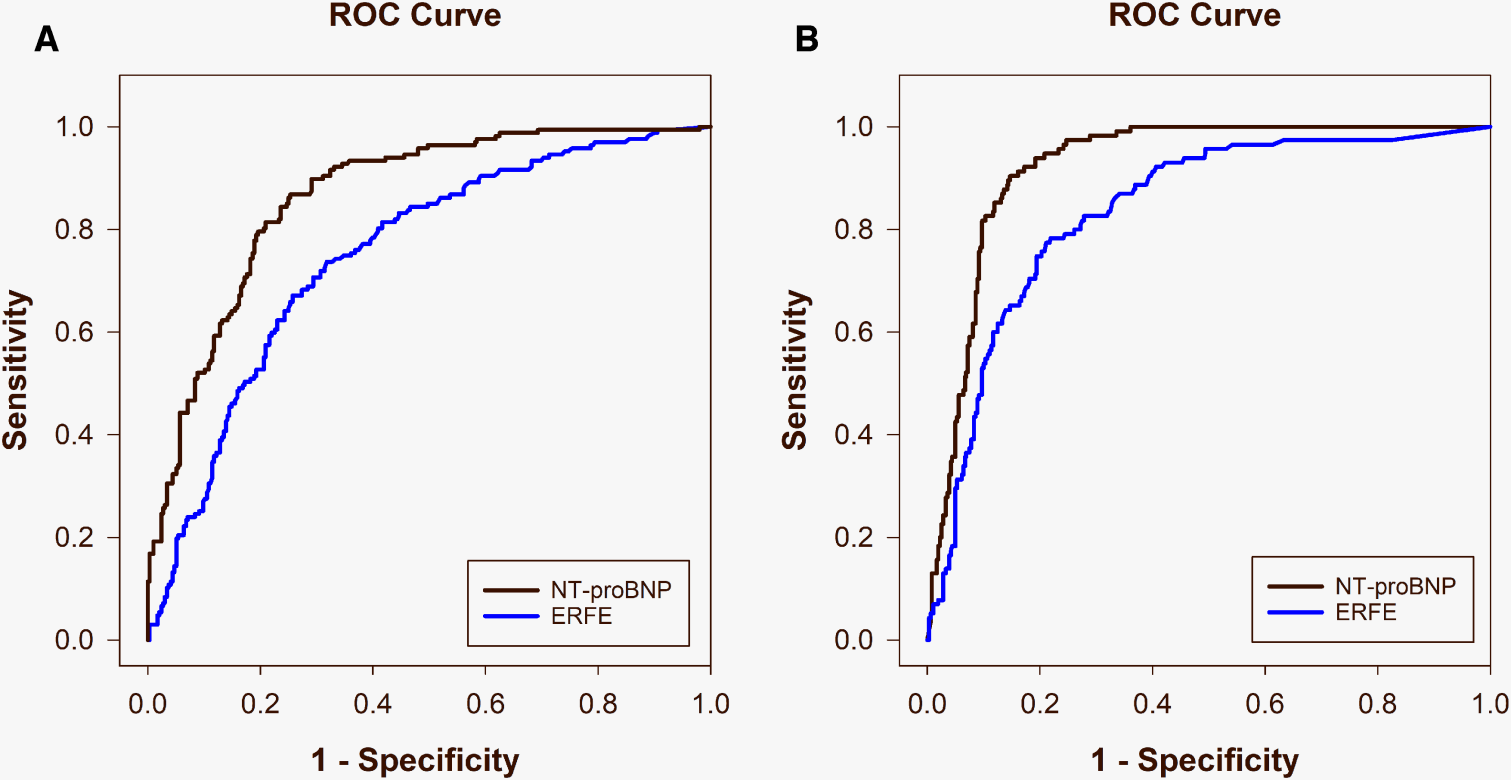
Area under the receiver operating characteristic curves comparing ERFE (blue) and NT-proBNP (black) for diagnosis of acute decompensated heart failure in the New Zealand [(**A**) ERFE: 0.75, NT-proBNP: 0.86] and Singapore [(**B**) ERFE: 0.84, NT-proBNP: 0.92] populations.

Median ERFE levels were elevated in ADHF in both cohorts compared with other diagnoses (NZ: 4.9 [2.4–11.0] ng/ml vs. 1.4 [0.6–3.2] ng/ml, *p* < 0.001 and SG: 4.2 [1.7–8.0] ng/ml vs. 0.4 [0.1–1.4] ng/ml, *p* = 0.021). Accordingly, logistic regression revealed ERFE to be independently associated with the diagnosis of ADHF in both the NZ and SG populations (both *p* = 0.003). This relationship was weaker than NT-proBNP which was more tightly associated with ADHF (*p* < 0.001 in both cohorts). Table 5 compares test performance characteristics for ERFE and NT-proBNP in the diagnosis of ADHF in the overall dyspneic population and the comorbid subgroups. In the discrimination of ADHF, ERFE performed strongly but was inferior to NT-proBNP. Both ERFE and NT-proBNP performed significantly better in the Asian cohort than the Western cohort (Figure 1 and Table 5): ERFE AUCs 0.84 vs. 0.75 (*p* = 0.006) and NT-proBNP AUCs 0.92 vs. 0.86 (*p* = 0.004). For ERFE, sensitivity and PPV were comparable between NZ and SG, respectively; whereas specificity, NPV and accuracy were better in SG compared to NZ (Table 5). NT-proBNP performed similarly to previously published data (12) in these two cohorts.

**Table 5:** Diagnostic performance of ERFE and NT-proBNP.

	ERFE (ng/ml)						NT-proBNP (ng/L)					
	All		AF		Obesity		All		AF		Obesity	
	NZ	SG	NZ	SG	NZ	SG	NZ	SG	NZ	SG	NZ	SG
*N*	479	475	105	44	150	164	463	475	102	44	145	164
Cut-off point	2.5	1.5	3.5	3.3	3.2	1.6	1539	829	2031	1405	946	723
AUC	0.75	0.84	0.71	0.61	0.79	0.87	0.86	0.92	0.70	0.64	0.87	0.94
95% CI	0.71–0.80	0.80–0.88	0.61–0.82	0.44–0.79	0.71–0.86	0.82–0.93	0.83–0.90	0.90–0.95	0.60–0.81	0.46–0.82	0.81–0.93	0.90–0.97
Sensitivity (%)	74	78	70	58	77	82	81	90	78	85	87	88
Specificity (%)	67	78	69	67	72	80	79	85	55	50	79	89
PPV (%)	56	53	79	71	62	63	69	66	75	71	72	77
NPV (%)	83	92	57	52	84	91	88	96	60	69	91	94
Accuracy (%)	70	78	70	61	74	80	80	80	70	70	82	88

AUC, area under the curve; CI, confidence interval; PPV, positive predictive value; NPV, negative predictive value; ERFE, erythroferrone; NT-proBNP, amino-terminal proBNP; NZ, New Zealand; SG, Singapore.

### 3.4 Subgroup analyses

As AF is known to impair the performance of NT-proBNP in diagnosing ADHF, we assessed whether the diagnostic performance of ERFE in the presence of AF may be impacted. In both cohorts, median ERFE levels were elevated in AF compared with non-AF (NZ: 4.1 [1.9–10.3] ng/ml, *n* = 105 vs. 2.0 [0.7–4.8] ng/ml, *n* = 349, *p* < 0.001; and SG: 2.9 [1.3–5.4] ng/ml, *n* = 44 vs. 0.7 [0.2–2.8] ng/ml, *n* = 392, *p* < 0.001). In univariate analysis for the discrimination of ADHF in the presence of AF, AUCs in both countries are reduced and similar (NZ; 0.71, SG; 0.61, *p* = 0.331), and not inferior to that of NT-proBNP (NZ; *p* = 0.968, SG; *p* = 0.728) (Table 5). Specificity was comparable for ERFE in both countries, whereas PPV, NPV and accuracy were slightly better and sensitivity substantially better in NZ than SG.

As obesity has also been shown to reduce the discriminatory power of NT-proBNP in diagnosing ADHF, we explored the impact of BMI on the performance of ERFE. Median ERFE levels were similar between patients with obesity and non-obese patients in both cohorts (NZ: 2.7 [0.99–7.8] ng/ml, *n* = 148 vs. 2.3 [0.81–5.1] ng/ml, *n* = 302, *p* = 0.579 and SG: 0.81 [0.22–3.6] ng/ml, *n* = 163 vs. 0.69 [0.19–2.8] ng/ml, *n* = 241, *p* = 0.902). Univariate analysis for the discrimination of ADHF in patients with obesity revealed ERFE to perform slightly better in the Asian cohort (AUC 0.87, *n* = 164) compared with the Western cohort (AUC 0.79, *n* = 150), but this was not significant (*p* = 0.066). NT-proBNP was significantly better than ERFE at diagnosing ADHF in SG (AUC 0.94, *p* = 0.027) but not NZ (AUC 0.87, *p* = 0.073). For ERFE, sensitivity, PPV and accuracy were comparable between countries, whereas specificity and NPV trended higher in SG than NZ (Table 5). The AUCs for ERFE and NT-proBNP in the subgroup of patients with obesity, were similar to those of the overall dyspneic population for both countries.

## 4 Discussion

We have measured circulating ERFE concentrations and assessed the ability of ERFE to diagnose ADHF among patients presenting to emergency departments in New Zealand and Singapore with acute breathlessness. The major findings from this work are (1) circulating ERFE levels were significantly elevated in ADHF versus all other diagnoses in both Western and Asian populations, (2) ERFE had moderate diagnostic power for ADHF but was outperformed by the current gold standard, NT-proBNP in both populations, (3) circulating ERFE was significantly elevated in patients with AF in both cohorts, (4) ERFE levels were not altered with obesity and (5) in the presence of AF the diagnostic performance of ERFE becomes comparable rather than inferior to that of NT-proBNP, whereas in patients with obesity, NT-proBNP is a superior predictor of ADHF in both countries.

To our knowledge, this is the first report on the diagnostic utility of ERFE for ADHF in an acutely breathless population. The differences in results seen between the NZ and SG populations could be explained in part by the different patient characteristics, whereby the Asian cohort was on average 17 years younger and had a lower burden of comorbidity compared to the Western cohort (12). Similar to NT-proBNP (12), ERFE was found to have better diagnostic performance for discriminating ADHF in the SG cohort compared with the NZ cohort. ERFE also had higher NPVs than PPVs, so like NT-proBNP, ERFE may be better at ruling out ADHF than ruling in. However, this was only seen in the overall dyspneic population and the subgroup of patients with obesity. In patients with AF, both ERFE and NT-proBNP had higher PPVs and lower NPVs.

The exact mechanism responsible for the increased plasma ERFE concentrations in HF patients is not clear. There are several possible explanations. First, ERFE levels increase to meet the demands of increased erythropoietic activity, which is stimulated by erythropoietin (EPO). In HF patients, EPO levels have been found to be elevated, which may explain the increase in ERFE production (14, 15). Second, our findings are in agreement with studies investigating the principal iron regulatory hormone, hepcidin, in HF. Contrary to most inflammatory diseases whereby hepcidin levels are high, hepcidin levels are known to be lower in patients with increasing HF severity as assessed by NYHA class (16). As ERFE is known to suppress hepcidin production, it may partly explain the lower concentrations of hepcidin in HF (7, 17). These possible explanations require follow up in further studies.

Although not tested in the present study, ERFE's involvement in iron regulation may mean that it has direct relevance to HF patients with anaemia, as iron deficiency is estimated to be present in up to 50% of chronic heart failure patients (18). These patients have a reduced exercise capacity and impaired health-related quality of life, a higher rate of 30-day re-hospitalisations and poorer prognosis with higher risk of major adverse cardiovascular events and almost double the risk of mortality (19, 20). In anaemic patients, ERFE may be able to complement or provide additional diagnostic information to NT-proBNP. Unfortunately, sample numbers preclude formal analyses on this subgroup and data on iron levels were unavailable for patients in this current study and future studies assessing this are required.

Our research has some limitations. First, there are a paucity of reliable assays to measure ERFE. We have used the Intrinsic LifeSciences ELISA which we have validated and proven to be the most reliable assay for ERFE measurement (21). The performance of this assay with respect to variability is not yet fully optimised, but even so, for the purpose of this study, circulating levels of ERFE were well-separated in ADHF compared with other diagnoses (4.9 vs. 1.4 ng/ml) allowing good discrimination of ADHF from non-ADHF phenotypes. This is in agreement with other validation studies of human ERFE assays, whereby although certain assay parameters had limitations, pathologies could be differentiated based on ERFE measurement (22). Second, whilst our sample size is satisfactory for deriving the diagnostic potential for ERFE, it requires validation in a larger, multicentre recruited set of patients with sufficient comorbid cases (e.g., AF, obesity) as well as accurate current iron and/or ferritin measurements. Further, as ERFE levels were different in the NZ and SG cohorts which may point to important ethnic differences, this warrants replication in other geographical settings.

In summary, we have demonstrated that, like NT-proBNP circulating ERFE is significantly higher in ADHF than in other causes of new onset breathlessness and higher in patients with AF, but unlike NT-proBNP, does not appear to be influenced by obesity. ERFE has fair diagnostic utility for ADHF, performing better in Asian than in Western patients. Although ERFE was unable to match NT-proBNP in discrimination of ADHF among the overall dyspneic population, ERFE was comparable in the sub-group of patients with AF, a condition which is well-documented to impair the diagnostic performance of NT-proBNP. We conclude that further investigations are required to fully understand the role of ERFE in patients with HF.

## Data Availability

The datasets presented in this article are not readily available because availability is dependent on study participant consent. Requests to access the datasets should be directed to sarah.appleby@otago.ac.nz.
