# Supplementary material for: Circulating erythroferrone has diagnostic utility for acute decompensated heart failure in patients presenting with acute or worsening dyspnea
Source: Front Cardiovasc Med. 2024 Jan 8;10:1195082. doi: 10.3389/fcvm.2023.1195082 (PMC10800458; doi:10.3389/fcvm.2023.1195082)
Supplement: Supplementary file 1 [file Table1.docx]

Supplementary table 1. Clinical characteristics of the subgroups of patients with atrial fibrillation and obesity

|  | **Atrial Fibrillation** |  |  | **Obesity** |  |  |
| --- | --- | --- | --- | --- | --- | --- |
|  | NZ (n=105) | SG (n=44) | P-value | NZ (n=148) | SG (n=163) | P-value |
|  | Median (IQR) | Median (IQR) |  | Median (IQR) | Median (IQR) |  |
| Age (years) | 77 (70-83) | 61 (53-72) | <0.001 | 69 (57-75) | 52 (45-61) | <0.001 |
| BMI (kg/m^2^) | 27.7 (24.1-32.9) | 26.5 (23.6-29.4) | 0.196 | 34.0 (31.6-38.4) | 31.1 (29.0-35.3) | <0.001 |
| eGFR (mL/min/1.73m^2^) | 54.0 (42.5-68.0 | 66.4 (45.6-86.3) | 0.016 | 60.0 (49.5-72.0) | 82.7 (63.7-101.6) | <0.001 |
| SaO_2_ (%) | 96.0 (93.5-97.0) | 98.0 (96.3-100.0) | <0.001 | 96.0 (94.0-97.3) | 98.0 (96.0-99.0) | <0.001 |
| Heart rate (bpm) | 104.0 (78.5-129.5) | 97.5 (82.3-129.3) | 0.680 | 89.5 (76.5-110.5) | 91.0 (76.0-103.0) | 0.136 |
| Haemoglobin | 131.5 (116.0-142.8) | 131.0 (120.0-147.5) | 0.183 | 137.0 (119.5-148.0) | 135.0 (120.5-151.0) | 0.846 |
| ERFE (ng/mL) | 4.1 (1.9-10.3) | 2.9 (1.3-5.4) | 0.012 | 2.7 (1.0-7.8) | 0.8 (0.2-3.6) | <0.001 |
| NT-proBNP (ng/L) | 3045.8 (1467.1-7281.7) | 2335.5 (872.2-5835.0) | 0.259 | 877.8 (245.7-2693.6) | 167.2 (32.2-1761.0) | 0.087 |
| *CRP (mg/L) | 20.5 (7.8-79.5) | ND |  | 20.5 (6.0-67.8) | ND |  |
| WBC (x10^9^/L) | 8.2 (6.6-10.9) | ND |  | 8.9 (7.2-12.0) | ND |  |
|  | **Count (%)** | **Count (%)** |  | **Count (%)** | **Count (%)** |  |
| Gender (female) | 37 (35.2%) | 11 (25.0%) | 0.222 | 65 (43.3%) | 55 (33.7%) | 0.081 |
| **ADHF** | 66 (62.9%) | 26 (59.1%) | 0.666 | 56 (37.3%) | 49 (30.1%) | 0.173 |
| **Medical history** |  |  |  |  |  |  |
| CHF | 55 (52.4%) | 12 (27.3%) | 0.005 | 55 (36.9%) | 26 (16.0%) | <0.001 |
| MI | 36 (35.0%) | 10 (22.7%) | 0.143 | 34 (23.1%) | 28 (17.2%) | 0.191 |
| CAD | 59 (57.3%) | 20 (45.5%) | 0.188 | 66 (44.9%) | 57 (35.0%) | 0.074 |
| Hypertension | 73 (70.0%) | 26 (59.1%) | 0.219 | 103 (69.1%) | 109 (66.9%) | 0.670 |
| CABG | 22 (21.0%) | 9 (20.5%) | 0.946 | 18 (12.0%) | 19 (11.7%) | 0.925 |
| PTCA | 16 (15.2%) | 5 (11.4%) | 0.535 | 27 (18.0%) | 21 (12.9%) | 0.209 |
| Diabetes | 20 (19.2%) | 14 (31.8%) | 0.096 | 55 (37.2%) | 69 (42.3%) | 0.352 |
| Hyperlipidaemia | 48 (46.6%) | 22 (50.0%) | 0.706 | 83 (56.5%) | 86 (52.8%) | 0.513 |
| Renal impairment | 26 (24.8%) | 8 (18.2%) | 0.383 | 32 (21.5%) | 14 (8.6%) | 0.001 |
| COPD | 29 (27.6%) | 2 (4.5%) | 0.002 | 46 (30.9%) | 4 (2.5%) | <0.001 |
| Liver disease | 4 (3.8%) | 2 (4.5%) | 0.835 | 9 (6.0%) | 6 (3.7%) | 0.337 |
| Cancer | 33 (31.4%) | 3 (6.8%) | 0.001 | 34 (22.8%) | 8 (4.9%) | <0.001 |
| Arrhythmia | 86 (81.9%) | 30 (38.2%) | 0.066 | 56 (37.6%) | 15 (9.2%) | <0.001 |
| **Symptoms and signs** |  |  |  |  |  |  |
| PND | 46 (46.5%) | 16 (36.4%) | 0.261 | 71 (48.6%) | 71 (44.1%) | 0.427 |
| Orthopnoea | 69 (67.0%) | 24 (54.5%) | 0.152 | 105 (71.9%) | 87 (54.0%) | 0.001 |
| Dyspnea at rest | 83 (79.0%) | 25 (56.8%) | 0.006 | 118 (78.7%) | 92 (56.4%) | <0.001 |
| Rales | 72 (69.2%) | 23 (52.3%) | 0.049 | 83 (56.1%) | 65 (39.9%) | 0.004 |
| Rhonchi | 9 (8.6%) | 3 (6.8%) | 0.720 | 17 (11.3%) | 28 (17.2%) | 0.141 |
| Oedema | 61 (58.1%) | 18 (40.9%) | 0.055 | 74 (49.3%) | 51 (31.3%) | 0.001 |
| **ECG findings** |  |  |  |  |  |  |
| Normal |  |  |  | 37 (26.1%) | 61 (40.1%) | 0.011 |
| Atrial fibrillation |  |  |  | 38 (26.8%) | 16 (10.5%) | <0.001 |
| LBBB | 7 (6.7%) | 0 (0%) | 0.079 | 10 (7.0%) | 4 (2.6%) | 0.078 |
| **Chest X-ray findings** |  |  |  |  |  |  |
| Normal | 12 (11.8%) | 8 (18.2%) | 0.301 | 39 (26.9%) | 76 (49.4%) | <0.001 |
| Interstitial oedema | 16 (15.7%) | 12 (27.3%) | 0.103 | 19 (13.0%) | 21 (13.6%) | 0.874 |
| Pneumonia | 11 (10.5%) | 0 (0%) | 0.026 | 16 (10.7%) | 3 (1.8%) | 0.001 |

*Data available for 66/105 for the AF subgroup and 92/148 for patients with obesity

ADHF, acute decompensated heart failure; BMI, body mass index; eGFR, estimated glomerular filtration rate; SaO_2_, arterial oxygen saturation; ERFE, Erythroferrone; NT-proBNP, amino-terminal proBNP; CRP, C-reactive protein; WBC, white blood cell count; CHF, chronic heart failure; MI, myocardial infarction; CAD, coronary artery disease; CABG, coronary artery bypass grafting; PTCA, percutaneous transluminal coronary angioplasty; COPD, chronic obstructive pulmonary disease; PND, paroxysmal nocturnal dyspnea; LBBB, left bundle branch block.
